# Nutritional and metabolic responses of sheep fed banana leaf hay treated with sodium hydroxide

**DOI:** 10.1007/s11250-026-04994-y

**Published:** 2026-03-24

**Authors:** Hélio Oliveira Neves, Dorismar David Alves, Fredson Vieira e Silva, Laura Lúcia dos Santos Oliveira, Luciana Castro Geraseev, Marielly Maria Almeida Moura, Gabriel Carvalho Rezende Velasquez Santos, Adriano Mendes Vasconcelos, Janiquele Soares Silva Batista, Lara Danieli Lopes Fernandes, Cléverton Lopes Lacerda, Gabriel Santos Souza David

**Affiliations:** 1 Instituto Federal do Norte de Minas Gerais, Salinas, MG Brazil; 2 Department of Agricultural Sciences, Universidade Estadual de Montes Claros, Reinaldo Viana Street, Janauba, MG 39448-524 Brazil; 3 Institute of Agricultural Sciences, Universidade Federal de Minas Gerais, Montes Claros, MG 39404-547 Brazil; 4 Department of Animal Science, Universidade Estadual Paulista, Jaboticabal, SP 14884-900 Brazil

**Keywords:** Alkaline, Intake, Ruminants, Fiber

## Abstract

Our objective was to evaluate the impacts of the treatment of banana leaf hay with levels of sodium hydroxide (NaOH) on intake, digestibility, feeding behavior, and blood parameters of sheep. Five ½ Santa Ines × Dorper sheep with body weight of 38.43 ± 4.38 kg were used in a Latin square design. Treatments were based on levels of NaOH in banana leaf hay, as follows: 0; 1.25; 2.5; 3.75 and 5%. Overall, the intakes of dry matter, organic matter, crude protein, and total digestible nutrients increased linearly (*P* < 0.001) as a function of the dose NaOH. NaOH treatment of leaf hay linearly increased (*P* ≤ 0.04) the digestibility coefficients of organic matter and fiber, with an increase of 11 percentage points in fiber digestibility. However, we did not observe (*P* ≥ 0.33) effect of NaOH on crude protein digestibility. No effect was observed (*P* ≥ 0.07) on uric acid, creatinine, cholesterol, aspartate aminotransferase or urea. Increasing the NaOH dose did not alter the eating, rumination, and idlling time of sheep (*P ≥* 0.06). However, ruminating efficiency for dry matter and fiber were increased (*P* < 0.02) as a function of the increased NaOH level. Chewing characteristics (i.e., number of bolus/day, time/bolus, and chewing/bolus) were not influenced by the NaOH levels (*P* ≥ 0.06). Chemical treatment of banana leaf hay with NaOH increased feed intake and fiber digestibility in sheep and it did not affect indicators of hepatic injury, while reducing blood urea concentration, suggesting improved efficiency of nitrogen utilization.

## Introduction

The gap between demand and supply of conventional feed resources for animal feeding across the world is increasing. To overcome this deficiency, studies of unconventional feed resources with potential application in ruminant feeding are necessary for the development of sustainable feed systems, and they must be available on a large scale and worldwide. Due to the large volume generated during banana harvesting, the use of waste for ruminants feed result in sustainable positive impacts.

In a study with dairy heifers, Rigueira et al. (2021) found crude protein (CP) contents of ~ 106 and 51.0 g/kg dry matter (DM) for banana leaf hay and pseudostem hay, respectively. This suggests a decrease in feed costs from the adoption of banana leaves in diets, due to the possibility of reduction protein concentrates in the diet. However, it has low fiber digestibility, which can reduce voluntary intake (Detmann et al. 2014). Indeed, it is common to observe a decrease in voluntary intake with the inclusion of banana leaves in the diet (Rigueira et al. 2021; Geraseev et al. 2025).

Although other banana by-products do not depress voluntary intake as much as the leaf (Geraseev et al. 2025), it seems to present a better balance between availability and operational complexity, since it does not require a large expenditure in terms of labor as the pseudostem and may be more accessible for ruminant feeding compared to the peel. Thus, improving their nutritional characteristics appears to be an interesting tool in ruminant nutrition.

Alkaline treatments have often been used (Chizzotti et al. 2015; Andrade et al. 2023) to maximize degradation in feeds with low fiber digestibility. Particularly, sodium hydroxide (NaOH) is one of the most efficient substances in the treatment of forages with low fiber digestibility (Ribeiro et al. 2009). However, it can cause a decrease in feed intake due to excess sodium (Lopez et al., 2021). Moreover, high levels of alkaline additives may affect the smell, palatability and osmolality in the rumen and have a corresponding negative impact on intake (Chizzotti et al. 2015). Therefore, it is important to study the nutritional responses of sheep to the treatment of banana leaf hay with NaOH.

Our hypothesis is that the treatment of banana leaf hay with NaOH reduces and disrupts some components of the cell wall of banana leaf hay, increasing apparent digestibility and voluntary intake, improving the feeding behavior, and maintaining the blood parameters. Thus, our objective was to evaluate the voluntary intake and apparent digestibility, blood parameters and feeding behavior of feedlot sheep fed banana leaf hay treated with NaOH.

## Materials and methods

### Animals, experiment design, and management

The experiment was carried out at the Universidade Estadual de Montes Claros (15° 49’ 51.001’’ S, 43° 16’10.443’’W), located in Janaúba, Minas Gerais, Brazil. All practices involving the use of animals were approved by the Institutional Animal Care Committee (Protocol no. 03/2016) of Universidade Estadual de Montes Claros. At the beginning of the experimental period, the animals were treated against endo- and ectoparasites.

The experiment lasted 70 days, divided into 5 periods of 14 days, 7 days for the adaptation of the animals to diets and 7 days for data collection and sampling. The experimental design was simultaneous in 5 × 5 Latin squares (5 diets, 5 experimental periods, and 5 animals). Five ½ Santa Ines × Dorper sheep were used (intact males), with an age of five months and body weight (BW) of 38.43 ± 4.38 kg. The animals were housed in individual pens (1 × 2 m) equipped with concrete floors, individual feeders, and water drinkers, which assured unrestricted access to water.

### Feeding and treatments

The treatments consisted of doses of 0, 1.25, 2.5, 3.75, and 5% sodium hydroxide applied on banana leaf hay (as-fed). The diets were composed of banana leaf hay and concentrate, with forage-to-concentrate ratio of 50:50 (DM basis). The chemical composition and ingredients of diets are present in Table 1. The experimental diets were formulated to be isoprotein and formulated in accordance with the recommendations of the National Research Council (2007), in order to meet the nutritional requirements of lambs with a weight of 38 kg and an estimated gain of 200 g/d. The feeding was performed twice a day (0700 h and 1600 h) to allow for *ad libitum* intake, allowing 100 g/kg in orts.

**Table 1:** Ingredient composition of experimental diets

Item	Levels of NaOH				
	0	1.25	2.5	3.75	5.0
*Ingredients in diet*,* g/kg*					
Banana leaf hay	489	486	480	477	476
Ground corn	426	425	425	421	419
Soybean meal	41.0	46.2	51.5	57.8	64.8
Calcitic limestone	26.0	26.0	25.9	25.7	25.3
Dicalcium phosphate	3.80	3.80	3.80	3.70	3.50
Mineral mix	5.60	5.60	5.70	5.70	5.70
Sodium bicarbonate	8.20	8.30	8.30	8.40	8.40
Monensin	5.00	5.00	5.00	5.00	5.00
*Chemical composition*,* g/kg DM*					
Dry matter, as fed	866	859	851	849	842
Organic matter	897	898	888	882	877
Crude protein	105	106	104	109	114
NDFap	425	402	408	387	373

NDFap: neutral detergent insoluble fiber corrected for ash and contaminant protein

The banana leaves were collected from a single banana plantation. The banana leaves were processed using a stationary forage chopper set to 20 mm, and then spread on a sun-exposed canvas, being manually turned once a day for drying. After drying, the banana leaf hay was treated with NaOH by spraying, using one liter of water per kilogram of as-fed banana leaf hay to dissolve the sodium hydroxide. After spraying, the material was again exposed to the open air for drying for five days and was then stored in bags.

## Sample collections

### Feed intake, apparent digestibility, water intake and urine output

In each experimental period, feed intake was quantified from day 8 to day 12, through the amount of diet offered from day 8 to day 12 and the orts collected from day 9 to day 13. Daily samples of ingredients and orts were collected and weighed individually for daily adjustment and calculation of feed intake. At the end of each experimental period, animals were weighed to calculate feed intake relative to BW.

Total fecal collections were performed from day 9 to day 13 of each experimental period, immediately after each spontaneous defecation, to calculate daily fecal output, to obtain digestibility of dietary compounds. Representative samples of feces (30%) and orts were collected, stored in plastic bags, and blended manually at the end of each period to obtain pooled samples per animal. Samples of diet ingredients were composed by period. All samples were dried in a forced air oven (55 °C) for 72 h and ground in a knife mill (Tecnal, 132 Piracicaba, SP, Brazil).

Water intake was quantified on the same days as feed intake, by weighing the water offered in plastic buckets and subtracting the leftovers after a period of 24 h. In this calculation, the water lost to evaporation daily was considered. For that, two plastic buckets with water were distributed in the same place as the stalls where water was offered and weighed every 24 h. In this same period, the output urine was recorded, through weighing buckets placed under the animals in metabolic stalls.

### Feeding behavior

Feeding behavior was assessed on day 13 of each period. Animals were visually observed for 24 h at 5-minute intervals, recording the time spent feeding, ruminating, and idle. Simultaneously, three observations were performed every 6 h for each animal to determine the number of chews per ruminal bolus and the time taken to ruminate each bolus. Rumination was expressed as minutes per kilogram of DM intake and minutes per kilogram of NDFap intake. Rumination efficiency was calculated according to the Bürger et al. (2000). The number of chews per ruminal bolus and the time spent chewing each bolus were recorded during the observation periods. Daily bolus ruminations were calculated by dividing the total rumination time (minutes) by the average time required to ruminate a bolus.

### Blood parameters

Blood samples were collected on day 14 of each trial period at 0700 h, previously the feeding. Samples were collected by jugular vein puncture, using vacuum tubes with a clot activator and gel for serum separation (BD Vacutainer^®^ SST^®^ II Advance^®^, São Paulo, Brazil) to quantity urea, uric acid, creatinine, aspartate aminotransferase, and cholesterol. A tube with EDTA and sodium fluoride (BD Vacutainer^®^ Fluorinated/EDTA, São Paulo, Brazil) was used to quantify the plasma glucose concentration. After collection, samples were centrifuged at 2200 × g for 20 min. Serum parameter analysis were performed using the enzymatic method for glucose and cholesterol, enzymatic-ultraviolet method for urea, enzymatic-colorimetric method for uric acid, colorimetric method for creatinine, and kinetic-ultraviolet method for aspartate aminotransferase.

### Laboratory analyses

Samples of ingredients, orts, and feces were analyzed. The analyzes performed were of dry matter (DM; dried over-night at 105 ◦C, method G-003/1), ash (method M-001/1), crude protein (CP; method INCT-CA N-001/1), and neutral detergent insoluble fiber (NDFap; using heat-stable alpha-amylase without sulfite and corrected for ash and protein; method F-002/1) method F-004/1) according to Detmann et al. (2012). Non-fibrous carbohydrates (NFC) were calculated as proposed by Detmann et al. (2012): NFC (g/Kg) = OM – (EE + NDFap + CP), where OM is the organic matter.

## Statistical analyses

The experiment was carried out and analyzed according to a 5 × 5 Latin square design, with five treatments, five animals, and five experimental periods, as follow:

$$Yijk = \mu + \tau {\rm{k}} + ai + pj + \varepsilon {\rm{ijk}},$$

where ${Y}_{ijk}$is the response observed for animal $i$, in period $j$ under treatment $k$; $\mu$is the overall constant; ${\tau}_{k}$is the fixed effect of treatment $k$; ${a}_{i}$is the random effect of animal $i$; ${p}_{j}$is the random effect of period $j$; and ${\epsilon}_{ijk}$is the residual error term, *N*(0, σ2).

When the effect of treatment (i.e., levels of NaOH) was significant, the linear, quadratic, and cubic effects were evaluated using orthogonal contrasts. The critical probability level for the occurrence of a Type I error was set at 0.05. Statistical analyses were performed using the MIXED procedure of SAS 9.4.

## Results

### Feed intake and digestibility

Overall, the intakes of dry matter, organic matter, crude protein, and total digestible nutrients increased linearly (*P* < 0.001; Table 2) as a function of the sodium hydroxide dose applied to the banana leaf hay. Dry matter intake increased by 220 g/d from the control treatment to the 5% NaOH treatment, representing an approximate 22% increase. The TDN intake of animals in the 0% NaOH treatment increased from 584 g/d to 753 g/d, corresponding to a rise of 169 g/d, or about 30%. In contrast, a cubic effect (*P* < 0.001) was observed for fiber intake (g/d), although only a linear effect (*P* < 0.01) was detected when fiber intake was expressed in g/kg BW.

**Table 2:** Effects of banana leaf treatment with sodium hydroxide on voluntary intake of confined sheep

Item	Levels of NaOH					SEM	*P*-value		
	0	1.25	2.5	3.75	5.00		L	Q	C
*g/d*									
Dry matter	1014	1051	1138	1227	1240	69.1	< 0.001	0.13	0.53
Organic matter	910	946	1014	1086	1094	62.1	< 0.001	0.22	0.66
Crude protein	107	112	121	136	141	8.1	< 0.001	0.072	0.78
NDFap	400	378	430	439	414	30.4	< 0.001	0.023	< 0.001
NFC	362	406	420	464	465	28.0	< 0.001	0.97	0.17
TDN	584	603	657	731	753	38.3	< 0.001	0.067	0.81
g/kg BW									
Dry matter	24.8	24.6	26.4	28.4	29.2	2.11	< 0.001	0.060	0.46
NDFap	9.4	9.4	9.9	10.2	10.0	0.76	0.009	0.50	0.23

NDFap: neutral detergent insoluble fiber corrected for ash and contaminant protein; NFC: non-fibrous carbohydrates; TDN: total digestible nutrientes; SEM = standard error of the mean; L = linear; Q = quadratic; C = cubic

Sodium hydroxide treatment of leaf hay linearly increased (*P* ≤ 0.042) the digestibility coefficients of organic matter and fiber, with an increase of approximately 11% points in fiber digestibility (Table 3). However, we did not observe (*P* ≥ 0.33) any effect of sodium hydroxide treatment of banana leaves on crude protein digestibility.

**Table 3:** Effects of banana leaf treatment with sodium hydroxide on digestibility of confined sheep

Item	Levels of NaOH					SEM	*P*-value		
	0	1.25	2.5	3.75	5.00		L	Q	C
g/kg DM									
Organic matter	605	619	614	638	650	1.5	0.041	0.42	0.094
Crude protein	537	526	523	539	577	1.8	0.94	0.33	0.82
NDFap	372	373	404	460	480	2.4	< 0.001	0.072	0.92

NDFap: neutral detergent insoluble fiber corrected for ash and contaminant protein; SEM = standard error of the mean; L = linear; Q = quadratic; C = cubic

### Blood parameters, water intake and urinary excretion

Increasing the sodium hydroxide dose applied to banana leaf hay promoted a linear increase (*P* < 0.05) in water intake and urinary excretion (*P* < 0.06) in sheep (Table 4). Water intake increased from 89.1 to 121 g/kg BW in the control treatment to 5% NaOH.

**Table 4:** Effects of banana leaf treatment with sodium hydroxide on blood parameters, urine output, and water intake of confined sheep

Item	Levels of NaOH					SEM	*P*-value		
	0	1.25	2.5	3.75	5.00		L	Q	C
*mg/dL*									
Glucose	55.8	59.8	58.5	63.6	62.7	2.32	0.015	0.75	0.15
Colesterol	70.6	69.2	68.4	65.6	64.2	2.43	0.67	0.69	0.75
Uric acid	1.40	1.80	1.74	1.70	1.44	0.272	0.46	0.39	0.67
Urea	24.5	22.0	21.8	21.4	17.5	2.22	0.07	0.33	0.58
Creatinine	1.22	1.34	1.16	1.02	1.10	0.106	0.09	0.19	0.43
AST, U/L	90.4	93.4	92.2	95.8	94.2	4.52	0.12	0.99	0.27
Urine output, mL/d	1090	964	1268	1324	1483	2845	0.049	0.42	0.20
Water intake, g/kg BW	89.1	94.6	99.8	111	121	17.0	0.05	0.72	0.86

AST: aspartate aminotransferase; SEM = standard error of the mean; L = linear; Q = quadratic; C = cubic

Serum creatinine levels of sheep were not affected (*P* ≥ 0.09) by increasing the sodium hydroxide dose applied to banana leaf hay (Table 4). Increasing the sodium hydroxide dose applied to banana leaf hay increased serum glucose levels in sheep (*P* < 0.02). However, no effect was observed (*P* ≥ 0.07) on uric acid, cholesterol, aspartate aminotransferase or urea.

### Feeding behavior

Increasing the sodium hydroxide dose applied to banana leaf hay did not alter the eating, rumination, and idlling time of sheep (*P* ≥ 0.06; Table 5). However, ruminating efficiency for dry matter and fiber were increased (*P* < 0.02) as a function of the increased sodium hydroxide dose. Chewing characteristics (i.e., number of bolus/day, time/bolus, and chewing/bolus) were not influenced by the NaOH doses (*P* ≥ 0.06).

**Table 5:** Effects of banana leaf treatment with sodium hydroxide on feeding behavior of confined sheep

	Levels of NaOH					SEM	*P*-value		
	0	1.25	2.5	3.75	5.00		L	Q	C
*Spent time*,* min/d*									
Eating	187	196	208	184	196	10.1	0.93	0.06	0.32
Ruminating	607	595	622	602	574	15.1	0.85	0.79	0.21
Iddling	660	661	601	667	681	23.7	0.59	0.072	0.030
*Ruminating efficiency rate*									
Dry matter	98.9	106	109	117	127	6.22	< 0.001	0.95	0.32
NDFap	39.6	39.3	41.5	44.0	45.3	2.82	0.016	0.27	0.69
*Chewing*									
Number of bolus/d	706	656	735	723	649	54.1	0.35	0.53	0.13
Time/bolus	52.8	54.9	52.5	53.1	48.5	4.34	0.83	0.61	0.25
Mastigation/bolus	70.9	71.1	71.6	66.6	69.9	4.56	0.067	0.085	0.38

NDFap: neutral detergent insoluble fiber corrected for ash and contaminant protein; SEM = standard error of the mean; L = linear; Q = quadratic; C = cubic

## Discussion

The use of alternative feed sources in sheep nutrition is important for the adequate disposal of by-products, agricultural production, and reduction of animal production costs. In this regard, the banana leaf has a high protein content (106 g/kg DM; Rigueira et al. 2021). On the other hand, it has low fiber digestibility which can reduce feed intake by ruminants (Allen et al. 2019; Detmann et al. 2014).

Alkaline treatments (e.g., sodium hydroxide and calcium oxide) have improved the nutritional value of feed for ruminants (Andrade et al. 2023). However, high levels of alkaline additives may affect the smell, palatability and osmolality in the rumen and have a corresponding negative impact on intake (Chizzotti et al. 2015). Indeed, excess sodium from sodium hydroxide can reduce feed intake (Lopez et al., 2021). Despite this, corroborating our hypothesis, there was no negative impact of NaOH on feed intake, demonstrating the potential for applying sodium hydroxide to banana leaves at levels of up to 5% (as-fed). Clearly, the animal adapted to the higher dietary sodium content through increased water intake, which avoided negative effects of sodium on ruminal osmolarity.

Banana leaf hay treatment with NaOH considerably increased voluntary intake (~ 22%). The increase in feed intake with the alkaline treatment can be attributed to the improvement in fiber digestibility (Detmann et al. 2014). Higher fiber digestibility reduces the rumen fill effect, thereby positively affecting feed intake and increasing the rate of passage (Waldo et al. 1972). Indeed, Rigueira et al. (2021), when evaluating hay from banana crop residues (leaf, bark, and pseudostem) as a replacement for sorghum silage in the diet of dairy heifers, observed lower feed intake in animals fed leaf hay and attributed this result to the lower fiber digestibility of that feed.

Moreover, the presence of tannins can affect diet palatability. Carmo et al. (2018) observed a high presence of tannins in banana leaves (7.80% of DM). Pereira Filho et al. (2003), analyzing the effect of treating forage hay with NaOH, reported a reduction in the tannin content in treated material. Thus, a possible decrease in tannin concentration may also have contributed to the improvement in feed intake. However, it should be emphasized that the main factor responsible for the increased intake is undoubtedly the greater fiber digestibility.

We observed a strong potential of sodium hydroxide to enhance the fiber digestibility of banana leaf hay, which increased by approximately 30% with the 5% NaOH treatment compared to the control (no alkaline treatment). An increase in fiber digestibility may be accompanied by increased microbial protein synthesis, which increases the supply of metabolizable protein to the intestine, thereby increasing animal performance (Detmann et al. 2024). Therefore, these results highlight the need for further studies evaluating the effects of sodium hydroxide treated banana leaf hay on animal performance, particularly given that this feedstuff has a relatively high crude protein content.

The increase in fiber digestibility observed is basically due to the action of sodium hydroxide on this nutritional component. Overall, alkaline treatments increase fiber digestibility in forages due to reduced crystallinity of the lignocellulosic structure (Chapple et al. 2015) and lead to the breakdown of hydrogen bonds in cellulose (Berger et al. 1994). Likewise, several authors found an increase in the digestible fraction of NDF with the inclusion of sodium hydroxide (Molina et al. 1983; Pina et al. 2009).

The cubic behavior observed in fiber intake can be attributed to a balance between the increase in dry matter intake and the reduction in neutral detergent fiber content with incresead of NaOH. A decrease in NDF intake was observed between 0 and 1.25% NaOH, which can be explained by the reduction in NDF concentration without a considerable increase in DMI. From 1.25 to 2.5% NaOH, the increase in NDF intake was substantial, mainly due to the marked rise in DMI, while the reduction in NDF content was less pronounced. However, at the 5% NaOH level, fiber intake decreased again, which can be attributed to a lower NDF intake. Indeed, a reduction in fibrous components with sodium hydroxide treatment has been reported previously (Pires et al. 2010). The decrease occurs because of the alkaline hydrolysis of plant cell wall constituents and cellulose swelling (Jackson 1977). Chizzotti et al. (2015), when evaluating the inclusion of calcium oxide in sugarcane silage for cattle, observed an increase in fiber intake, even though no differences were detected in feed intake. The increase of TDN intake reflects the increase in digestibility and intake of dry matter, as a function of NaOH levels. Additionally, the increase in crude protein intake was basically due to the increase in DM intake, since the diets were isoprotein.

We expected greater crude protein digestibility with alkaline treatment due to its high tannin content (Carmo et al. 2018). There are reports that sodium hydroxide can reduce tannin in feedstuffs (Pereira Filho et al. 2003). These tannins are water-soluble phenolic polymers, originating from the secondary metabolism of plants that complex with feed protein and may interfere with the digestibility of crude protein in the digestive tract (Van Soest 1994). Furthermore, banana leaves, in the study by Carmo et al. (2018), presented 4.90% of nitrogen insoluble in neutral detergent, a value higher than that of *Cynodon* ssp. and pseudostem. Some disruption of the fiber-protein bond could have occurred, which was not verified. Nevertheless, any effect found on protein digestibility could be confounded by increased crude protein intake, reflected in dilution of the fecal metabolic fraction.

Sodium hydroxide contains a high sodium concentration (Pires et al. 2010), which explains the increased urinary excretion and water intake observed in the animals. There is a positive association between dietary sodium content, sodium intake, urinary output, and water intake in cattle (Spek et al. 2012). As already mentioned, increased water intake is possibly a way for the animal to adjust ruminal osmolarity, which explains the absence of any effect of sodium hydroxide on voluntary intake.

Studies suggest that the increase in urinary volume, which can be induced by higher sodium levels in the diet, leads to greater sodium deposition in the soil. This accumulation may reduce nitrification and ammonia volatilization rates, thereby decreasing nitrous oxide emissions (Cardoso et al., 2019).

Although not statistically significant (*P* = 0.07), there was a trend for a linear reduction in serum urea concentration in animals of the alkaline treatment. Classically, a strong positive association exists between ruminal ammonia nitrogen and blood urea levels (Li et al. 2019). A reduction in blood urea concentration is an indication of greater efficiency in the utilization of nitrogen by rumen microorganisms (Gonçalves et al. 2025), suggesting greater microbial production. This finding is strongly supported by the higher protein and non-fibrous carbohydrates intakes observed in the animals. To achieve maximum ruminal efficiency in terms of microbial protein synthesis, a synergistic relationship between the rate of protein degradation, ammonia concentration, and carbohydrate fermentation is required (Van Soest 1994). Studies conducted in tropical regions have indicated that balanced protein–energy nutrition can promote interactive effects on nitrogen metabolism and improve animal performance (Palma et al. 2023). Once again, this finding reinforces the need for further research evaluating animal performance using this alkaline treatment technology applied to banana leaf hay.

The linear effect of sodium hydroxide treatment on serum glucose concentration can be explained by the higher dry matter intake observed in the animals, which results in an increased supply of gluconeogenic precursors. The levels of aspartate aminotransferase (AST) in serum can help people diagnose body tissues especially the heart and the liver are injured or not (Huang et al. 2006). Serum levels of AST did not differ among treatments, suggesting no detrimental effect of sodium hydroxide on liver function. Indeed, Oliveira et al. (2013), evaluating the effect of *Jatropha curcas* seeds treated with NaOH on liver function in dairy cows, found that the inclusion of up to 10% treated seeds in the diet did not negatively affect hepatic function. Reference values for AST in sheep are reported to range from 60 to 280 U/L (Radostits et al., 2002).

With the increase in fiber digestibility through sodium hydroxide treatment, pronounced changes in feeding behavior were expected; however, no major modifications were observed, except for the increase in ruminating efficiency. Ruminating efficiency of dry matter increased due to the reduction in fiber content with the alkaline treatment, combined with the elevated feed intake.

Chemical treatment of banana leaf hay with up to 5% sodium hydroxide markedly increases feed intake and fiber digestibility in sheep. Furthermore, it does not affect indicators of hepatic injury, while reducing blood urea concentration, suggesting improved efficiency of nitrogen utilization.

## Data Availability

The dataset is available upon reasonable request from the corresponding author.
